# Transcriptional regulation of mTORC1 in cancer

**DOI:** 10.18632/oncotarget.26229

**Published:** 2018-12-04

**Authors:** Chiara Di Malta, Andrea Ballabio

**Affiliations:** Andrea Ballabio: Telethon Institute of Genetics and Medicine (TIGEM), Pozzuoli, Naples, Italy; Jan and Dan Duncan Neurological Research Institute, Houston, Texas, USA; Department of Molecular and Human Genetics, Baylor College of Medicine, Houston, Texas, USA; Medical Genetics Unit, Department of Medical and Translational Science, Federico II University, Naples, Italy; Chiara Di Malta: Telethon Institute of Genetics and Medicine (TIGEM), Pozzuoli, Naples, Italy; Medical Genetics Unit, Department of Medical and, Translational Science, Federico II University, Naples, Italy

**Keywords:** TFEB, mTORC1, RagD, transcription, cancer, Autophagy

The mechanistic Target Of Rapamycin Complex 1 (mTORC1) is a fine regulator of cell metabolism and its oncogenic activation sustains cancer cell growth, survival and proliferation. mTORC1 is activated by Rheb on the lysosomal surface where it is recruited by nutrient-activated RagGTPases (Rags). Mammals have four Rags: Rag A, B, C and D, which form obligate RagA /RagB and RagC /RagD heterodimers [1].

The post-translational control of mTORC1 activity has been extensively studied, however, little is known about its transcriptional regulation. The basic helix-loop-helix MiT/TFE transcription factors (TFs) are master regulators of lysosomal and melanosomal biogenesis and autophagy. MiT/TFE family members bind to identical DNA sequences and regulate overlapping sets of genes. We and others have previously shown that mTORC1 negatively regulates this family of transcription factors. When nutrients are available mTORC1 phosphorylates MiT/TFE TFs leading to their cytoplasmic retention. Starvation and physical exercise promote TFEB and TFE3 nuclear translocation by either inhibiting mTORC1, or activating the phosphatase calcineurin, respectively [2].

Our recent study demonstrated that MiT/TFE TFs are major regulators of mTORC1 activity in response to nutrients, unveiling the existence of a feedback mechanism crucial for cell metabolism. We observed that upon nutrient stimulation overexpression of TFEB or TFE3 increased mTORC1 activation both *in vitro* and *in vivo*, whereas their depletion significantly impaired mTORC1 signaling and protein synthesis. This suggested that nutrient-dependent activation of mTORC1 is transcriptionally regulated by MiT/TFE TFs.

Physical exercise followed by a protein meal activates mTORC1 signaling, thus promoting protein synthesis and muscle growth [3]. We showed that mTORC1-dependent protein synthesis after exercise is significantly impaired in muscle-specific TFEB KO mice compared with control mice, demonstrating that MiT/TFE transcription factors are, at least in part, responsible for this effect.

These data led us to investigate the mechanisms by which MiT/TFE TFs regulate mTORC1. To this end, we tested whether genes controlling mTORC1 activity were transcriptional targets of MiT/TFE factors. We discovered that the transcript levels of the GTPase *RagD* were significantly downregulated upon TFEB silencing and upregulated after TFEB overexpression both *in vitro* and *in vivo*. Interestingly, *RagC* transcript levels, as well as those of *Folliculin* (*FLCN*), a GTPase activating protein (GAP) for RagC/D [1], were also regulated by MiT-TFE genes, albeit to a lesser extent compared to *RagD*. Chromatin immunoprecipitation confirmed that TFEB binds to the *RagD* promoter. Importantly, genome editing of the *RagD* promoter region bound by MiT/TFE factors strongly impaired mTORC1 signaling. Immunofluorescence and biochemical analysis revealed that mTORC1 recruitment to the lysosome increased in TFEB-overexpressing cells and decreased in TFEB-depleted cells or in cells lacking the *RagD* promoter region bound by MiT/TFE factors. Based on these data we concluded that MiT/TFE transcription factors control mTORC1 lysosomal localization and activation through transcriptional regulation of RagD GTPase.

Nutrient depletion and physical exercise promote MiT/TFE nuclear translocation and this leads to enhanced expression of RagD. Induction of RagD GTPase promotes mTORC1 lysosomal recruitment once nutrients become available to efficiently switch between catabolic and anabolic pathways.

Overexpression of either *TFE3* or *TFEB*, as a result of chromosomal translocation, was detected in patients affected by renal cell carcinoma (RCC) [4]. In addition, increased nuclear translocation of TFEB, TFE3 or MITF was found in pancreatic ductal adenocarcinoma (PDA) [5]. Finally, 30 to 40% of melanomas harbor *MITF* amplifications, whereas some patients carry missense mutation in *MITF* coding sequence [6]. These finding indicate that MiT-TFE TFs may act as oncogenes, however, the oncogenic pathways downstream MiT/TFE activation are still largely unknown.

Recently, our laboratory has reported activation of WNT-β catenin signaling in a mouse model of RCC induced by kidney-specific TFEB overexpression [7]. However, since MiT/TFE TFs regulate a large cohort of genes, involved in a variety of processes, the most realistic scenario is that they drive oncogenesis through multiple mechanisms. Interestingly, increased mTORC1 activity was reported in patients presenting TFE3-fusion RCC and the use of mTOR inhibitors resulted in partial suppression of cancer growth [4].

We hypothesized that RagD-mediated mTORC1 hyper-activation accounts, at least in part, for the oncogenic role of MiT/TFE transcription factors. Consistently, we found that patient-derived cell lines from melanoma, RCC and PDA associated with hyper-activation of MiT/TFE factors presented a significant increase in both RagD transcript levels and mTORC1 signaling. To evaluate the contribution of RagD overerexpression to MiT/TFE dependent tumor growth, we performed xenotransplantation experiments. A patient-derived melanoma cell line presenting high levels of MITF (501Mel) was infected with shRNA for RagD or control shRNA (Sh-Luc) and then transplanted in immuno-deficient mice. We observed that RagD silencing virtually abolished xenograft tumor growth of melanoma cells in mice, suggesting that RagD is a potent driver of MiT/TFE associated cancers.

**Figure 1:**
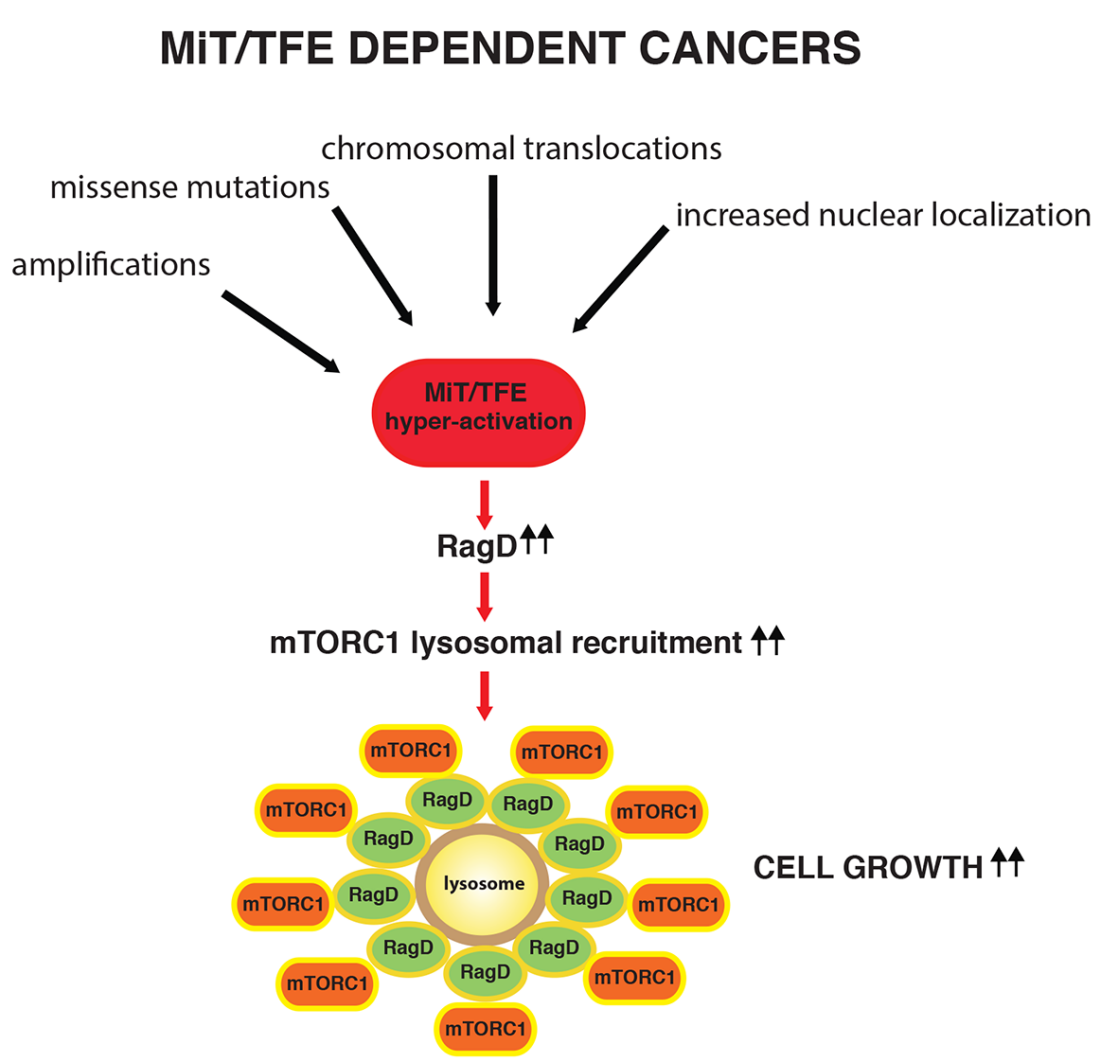
Model of mTORC1 hyperactivation in MiT/TFE dependent tumors MiT/TFE family members can be deregulated by genomic insults such as amplification, chromosomal translocations and missense mutations. In these cases, MiT/TFE Tfs are hyperactive causing constitutive RagD GTPase transcriptional induction, enhanced lysosomal recruitment and hyper-activation of mTORC1, which promotes cancer cell growth.

In conclusion, we have identified a novel regulatory pathway of mTORC1 signaling, centered on transcriptional induction of RagD GTPase. Malignancies associated to MIT/TFE hyper-activation result in constitutive RagD GTPase transcriptional induction and enhanced mTORC1 signaling which fuels tumor growth (Figure 1).

The discovery of this new oncogenic pathway can be relevant for therapy. Clinical data indicate that MiT/TFE-associated tumors show poor responsiveness to traditional chemotherapy treatments [6]. RagD transcript levels could represent a valuable biomarker for MiT/TFE dependent tumors to predict their responsiveness to treatment with mTOR inhibitors.
